# Supplementary material for: Complex‐centric proteome profiling by SEC‐SWATH‐MS
Source: Mol Syst Biol. 2019 Jan 14;15(1):e8438. doi: 10.15252/msb.20188438 (PMC6346213; doi:10.15252/msb.20188438)
Supplement: Supplementary file 7 — Dataset EV6 [file MSB-15-e8438-s007.zip › feature_plots_bioplex/Q9BV68.pdf]

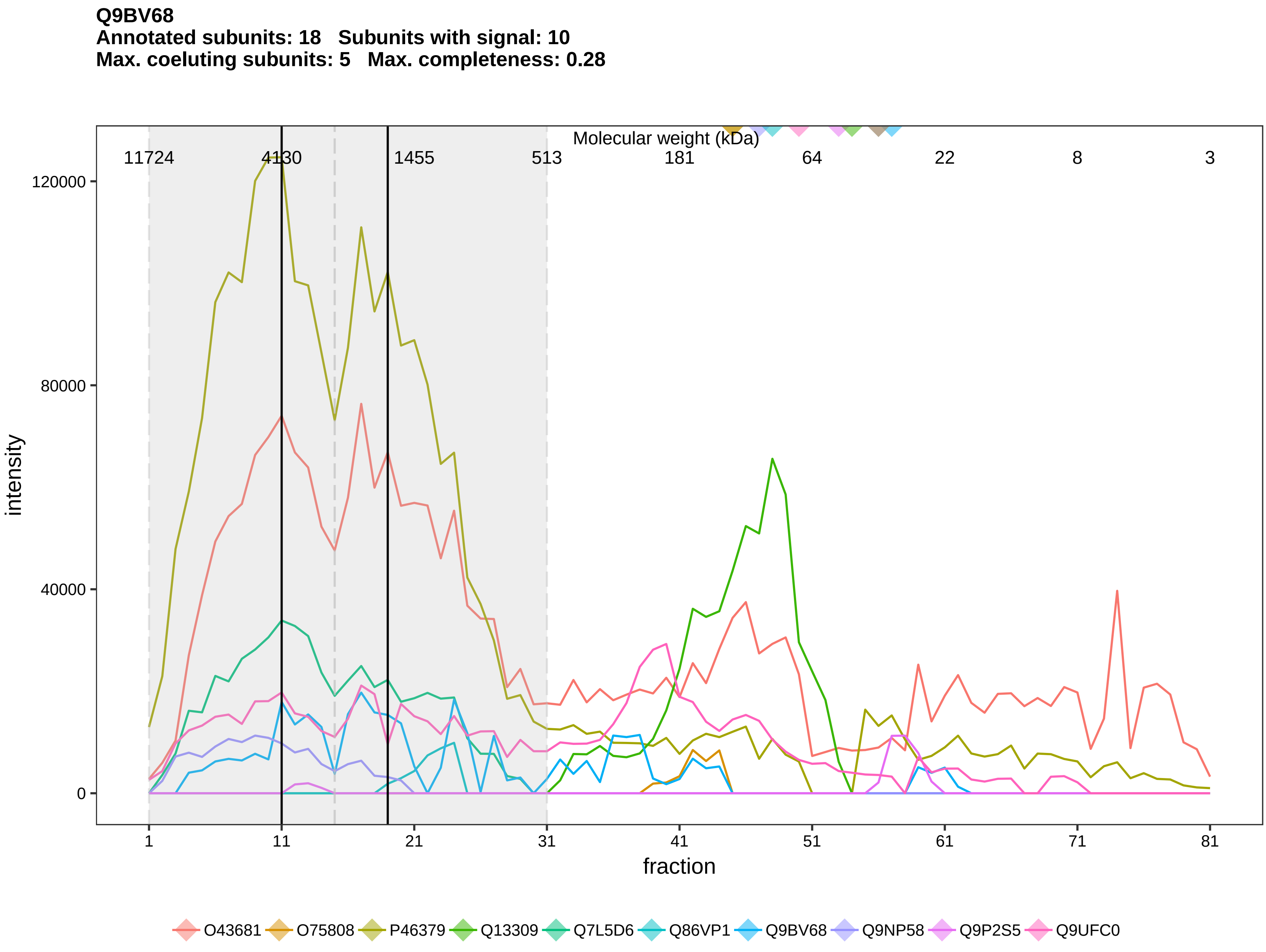
Q9BV68
Annotated subunits: 18   Subunits with signal: 10
Max. coeluting subunits: 5   Max. completeness: 0.28
Molecular weight (kDa)
11724
4130
1455
513
181
64
22
8
3
intensity
120000
80000
40000
0
fraction
1
11
21
31
41
51
61
71
81
O43681
O75808
P46379
Q13309
Q7L5D6
Q86VP1
Q9BV68
Q9NP58
Q9P2S5
Q9UFC0
